# Laser-assisted Excision of Gingival Overgrowth in an Endodontic Perforation: A Case Report

**DOI:** 10.7759/cureus.4378

**Published:** 2019-04-03

**Authors:** Deepak M Ravindran, Ram Sabarish, Devi Arul, Supraja Ajit, Dhivya M Harini

**Affiliations:** 1 Periodontology, Sri Ramachandra Medical College and Research Institute, Chennai, IND

**Keywords:** gingival polyp, gingival enlargement, diode laser, mineral trioxide aggregate (mta), endodontic perforations

## Abstract

With the increase in preventive and restorative dentistry, there is also an increase in the iatrogenic conditions that occur in modern dental practice. The goal of modern dentistry is to provide patients with a holistic solution by providing functional restoration. This case report will highlight one such case where a tooth was diagnosed as having a gingival overgrowth through a perforation during prior endodontic treatment. Proper diagnosis and treatment planning helped restore a tooth that would have been lost.

## Introduction

The aim of dentistry today is to salvage any tooth destroyed by dental caries, periodontal breakdown, or trauma. Modern technology allows the clinician to achieve that. However, there are times when overzealous treatment and the lack of skill and practice of the clinician causes more harm than good. One such iatrogenic complication in dentistry is endodontic perforations seen during root canal therapy. Accidental perforations are a serious complication of conservative therapy and can cause pain, abscesses, fistulae, and possible proliferation of gingival epithelium, especially if it occurs in the crestal area [1]. With a thorough diagnosis, treatment planning, and the use of modern restorative materials, a good clinical outcome can be achieved.

## Case presentation

A 33-year-old woman reported to the Department of Periodontology, Sri Ramachandra Dental College, Sri Ramachandra Institute of Higher Education & Research (DU), with the chief complaint of a missing tooth and the inability to masticate. She had no relevant medical history and had a history of undergoing orthodontic treatment, extraction of the lower left first molar, and endodontic treatment done three years back in relation to the left lower second molar. The patient had presented with fair oral hygiene.

**Figure 1:**
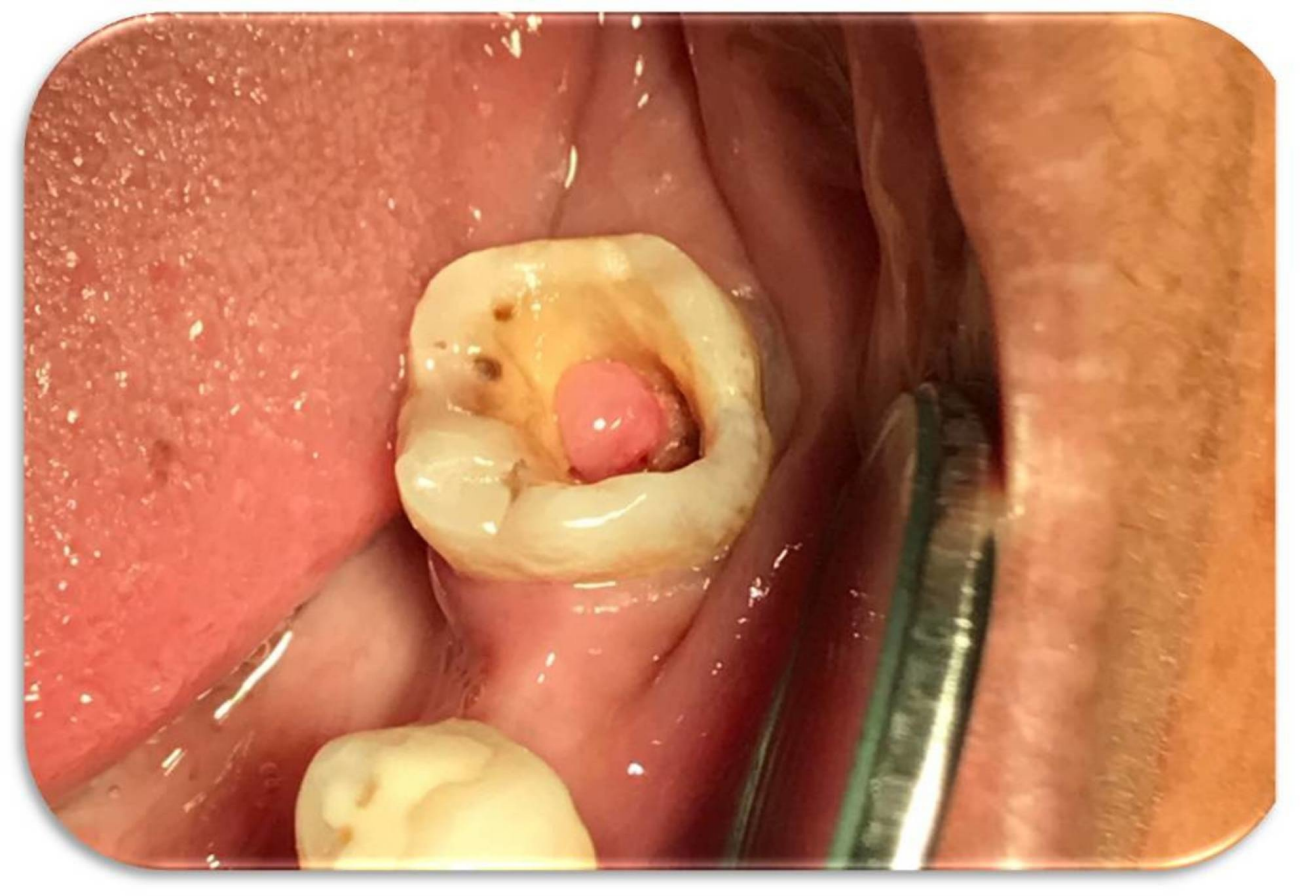
Preoperative

On clinical examination, a gingival growth was present on the floor of the endodontically treated tooth (#37), as seen in Figure 1.

**Figure 2:**
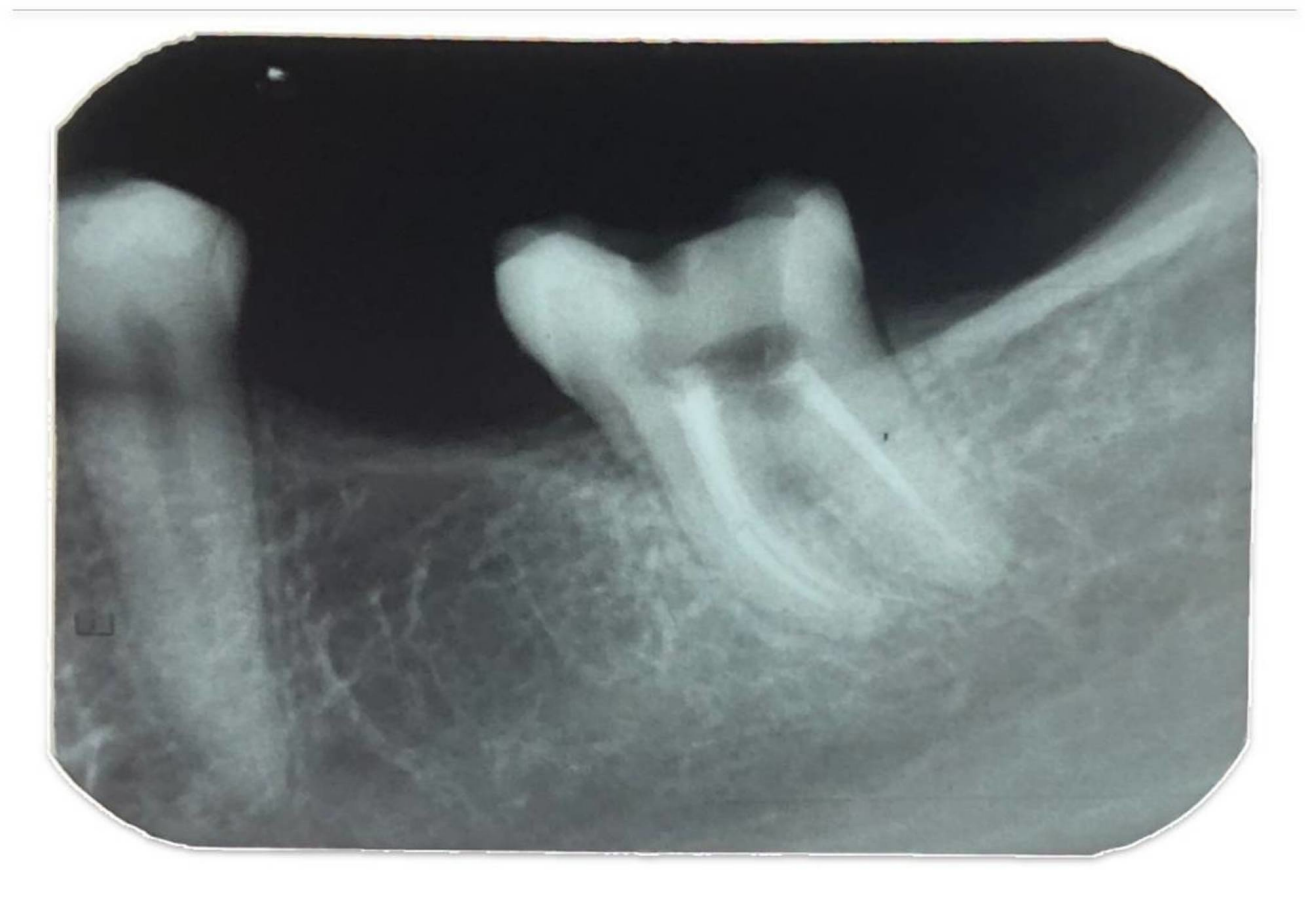
Preoperative Intraoral Radiograph

The tooth had no pain, probing pocket depth, and mobility associated with it. A provisional diagnosis of a gingival polyp was given. An intraoral periapical radiograph was taken, and it revealed no crestal bone loss or bone loss involving the furcation. However, radiolucency was seen on the distal surface of the tooth near the cementoenamel junction (Figure 2).

To substantiate the radiographic and clinical features and to establish the pathway of the polyp, a periodontal probe was inserted horizontally from the lingual aspect of the tooth and a small perforation was noticed on the distolingual aspect of 37. Furthermore, a Gutta Percha Point was inserted from the distolingual aspect and a pathway was established from the lingual marginal gingiva to the floor of the cavity.

A final diagnosis of gingival enlargement - gingival overgrowth due to accidental perforation was established. Treatment options included the extraction of the said tooth or the preservation of the tooth with a combination of periodontal procedures and endodontic materials, though it had a questionable prognosis. Both options were explained to the patient and the patient was willing to save the natural tooth, as she had extracted the tooth mesial to it.

**Figure 3:**
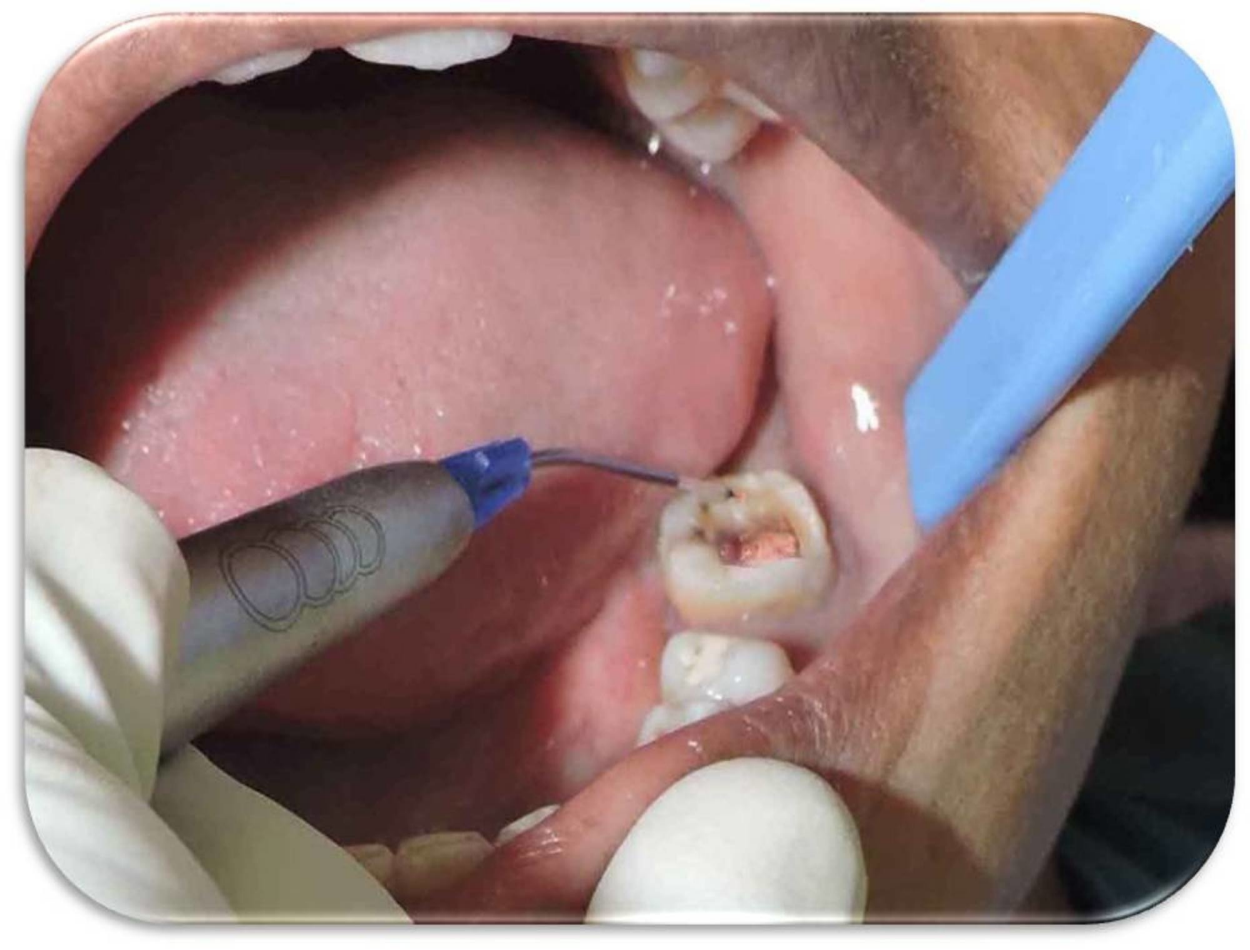
Laser-assisted Excision of the Growth

The treatment plan included laser-assisted excision of the polyp followed by the restoration of the perforation with mineral trioxide aggregate (MTA). After obtaining informed consent from the patient, initial cause-related treatment was carried out, following which the patient was taken up for the procedure. After sufficient anesthesia was given, laser-assisted excision was carried out with a diode laser (BioLase ezlaseTM 940; BioLase, CA, US), as shown in Figure 3.

**Figure 4:**
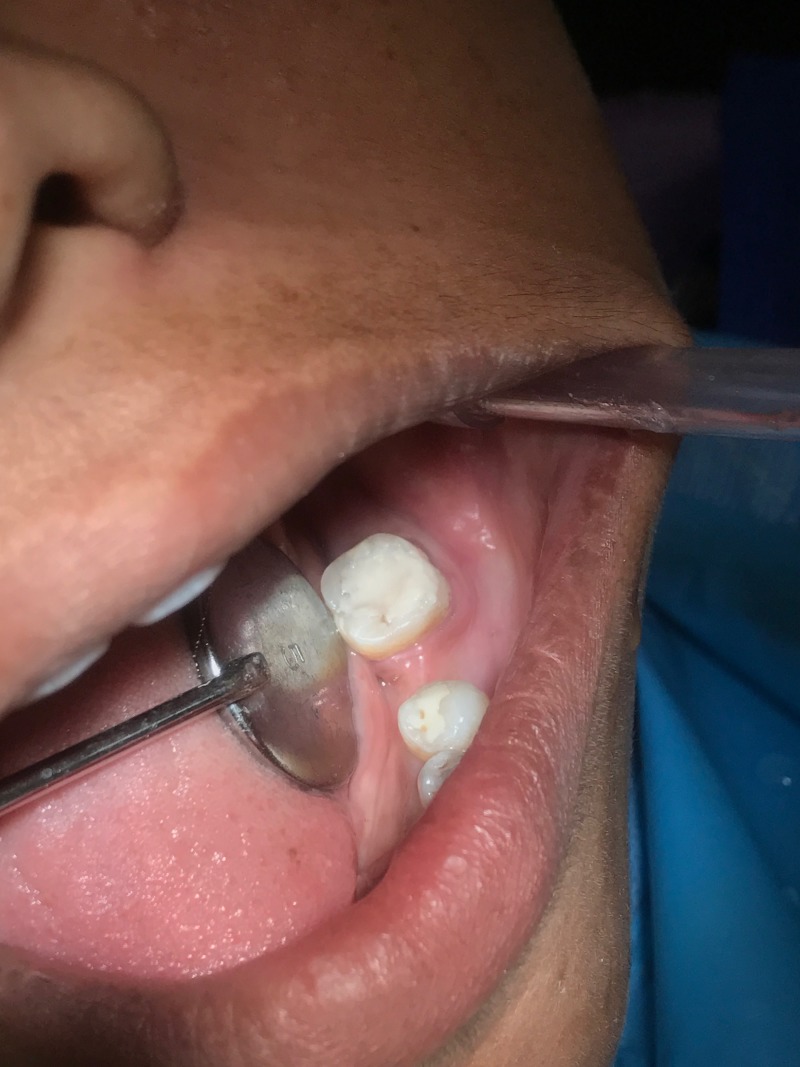
Final Postoperative

The excision was carried out in contact mode, with universal protocols from the lingual aspect of the tooth. After excision of the polyp, the perforation was checked for any tissue remnants. As there was no significant postoperative bleeding, the perforation was sealed using MTA. The sealant was mixed and placed in the perforation as per manufacturer’s instructions and restored with Glass Ionomer Cement - Type II (GC Fuji®, Tokyo, Japan) (Figure 4) [2].

**Figure 5:**
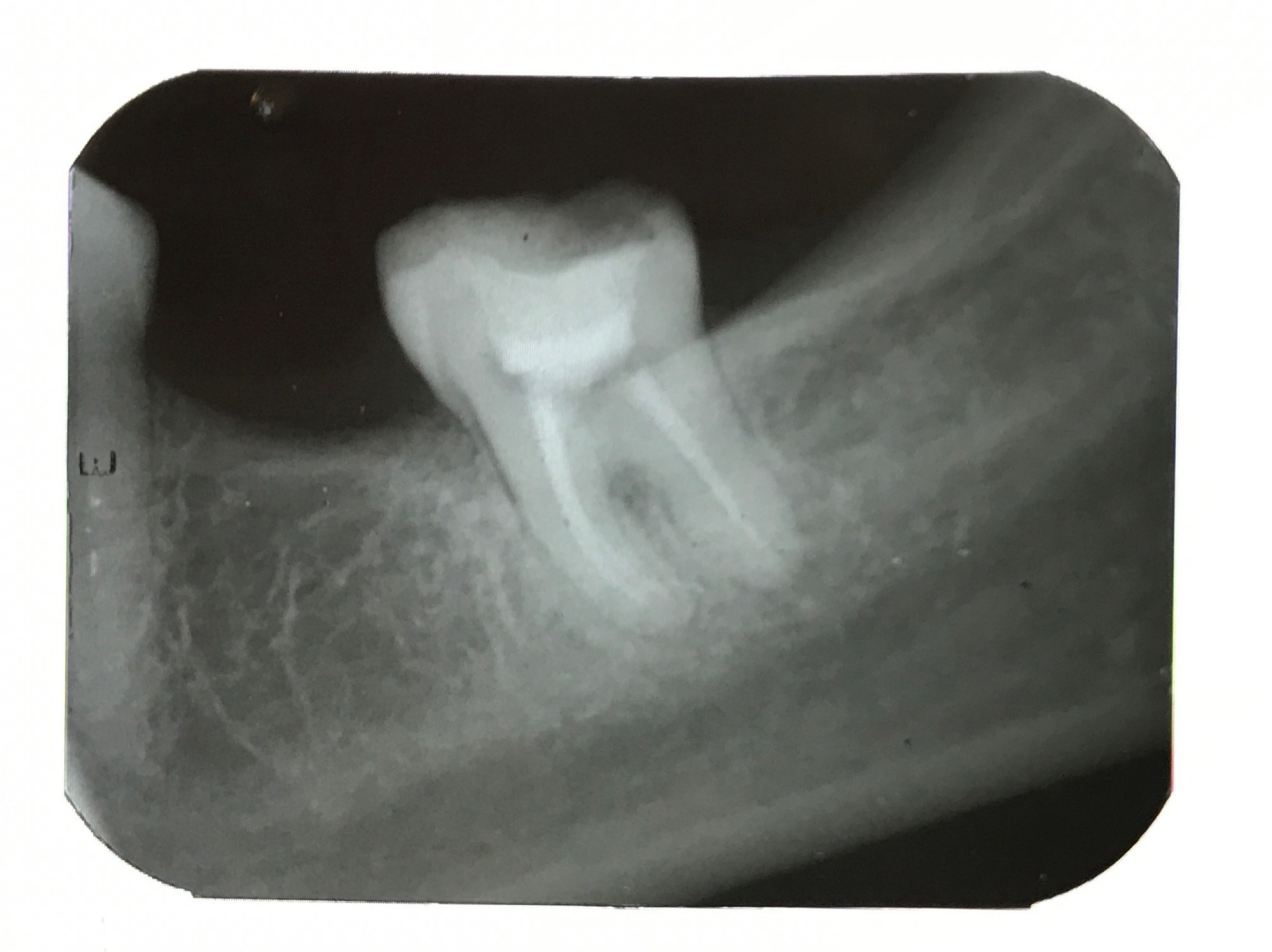
Postoperative Intraoral Radiograph

A periapical radiograph was taken to confirm the seal postoperatively (Figure 5).

No antibiotics or analgesics were prescribed postoperatively. Oral hygiene instructions were given to the patient.

The patient was reviewed the following day, and one week later, and the patient had no complaints or discomfort associated with the procedure. The patient was reviewed three months postoperatively and was asymptomatic and referred for further prosthodontic rehabilitation.

## Discussion

The goal of modern dentistry is to restore the tooth and periodontium to its normal form and function. In the above case, accidental perforation during endodontic treatment and the patient’s inability to continue dental treatment worsened the condition of the tooth. In sequence, careful clinical and radiographic examinations were performed to identify the problem and develop an accurate decision-making process to treat the iatrogenic perforation and subsequent gingival growth. As a result of this, an optimal treatment outcome was achieved.

Iatrogenic perforations occur in 2%-12% of all endodontically treated teeth [3]. The surprising feature, in this case, is that the perforation led to gingival growth from the floor of the cavity. One of the most important parameters affecting the prognosis is the location of the perforation. Based on the classification of root perforations [4], this case was considered a coronal perforation, wherein the perforation is coronal to the level of the crestal bone, there is epithelial attachment, with minimal damage to the supporting tissues, and easy access with a good prognosis. Though various options are available for the excision of the overgrowth such as scalpel or electrocautery, laser-assisted excision was preferred because less postoperative bleeding decontaminates the operating field, provides less operating time, and allows effortless excision [5-6].

Mineral trioxide aggregate (MTA) is a unique and versatile material in today’s dental practice. MTA has shown excellent results in pulp capping, pulpotomy, periapical surgery, as well as an excellent potential for apexogenesis and apexification [7]. MTA was chosen as the sealant for the perforation because of its superior marginal adaptation and sealing ability, higher regenerative potential, and because it allows the growth of periodontal ligament on its surface [8].

## Conclusions

Contemporary dental treatment must result in true oral health, incorporating comfort, function, and aesthetics. Clinical and radiographic evaluation, along with modern treatment modalities, helped conserve the tooth and provide a stable and functional result.
